# Tenosynovial Giant Cell Tumor (TGCT) in the Hip Joint With an *APOB* Mutation

**DOI:** 10.1155/cro/4675478

**Published:** 2026-05-15

**Authors:** Yujia Wang, Shaohua Zhang, Shuqiong Ge, Hongrui Zheng, Hanlong Xin

**Affiliations:** ^1^ Department of Orthopedics and Traumatology, Xianju County Traditional Chinese Medicine Hospital, Xianju, Zhejiang, China; ^2^ Department of Critical Care Medicine, Taizhou Hospital of Zhejiang Province Affiliated to Wenzhou Medical University, Linhai, Zhejiang Province, China, wmu.edu.cn; ^3^ Department of Gastroenterology, Taizhou Hospital of Zhejiang Province Affiliated to Wenzhou Medical University, Linhai, Zhejiang, China, wmu.edu.cn; ^4^ Department of Orthopedics, The First People′s Hospital of Wenling, The Affiliated Wenling Hospital of Wenzhou Medical University, Wenling, Zhejiang, China; ^5^ Department of Orthopedic Surgery, Taizhou Hospital of Zhejiang Province Affiliated to Wenzhou Medical University, Linhai, Zhejiang, China, wmu.edu.cn

## Abstract

A diffuse‐type tenosynovial giant cell tumor (D‐TGCT) is a rare, locally invasive tumor that mainly involves large weight‐bearing joints, such as the knees, hips, and ankles. Herein, we report a rare case of D‐TGCT of the hip joint in a 14‐year‐old girl. The patient presented with pain and limited movement without a history of trauma. Owing to the continuous progression of hip synovitis, whole exome sequencing was performed, which identified pathogenic variations in the frizzled class receptor 4 (NM_012193.3:c.379C>T(p.Arg127Cys)) and apolipoprotein B (NM_000384.2:c.10579C>T(p.Arg3527Trp)) genes. APOB mutations are associated with hypercholesterolemia. Magnetic resonance imaging of the right hip joint showed thickening of the synovial membrane with irregular edges, low signal intensity on T1‐weighted images, and high signal intensity on PDWI+FS images. Small nodules with low signal intensity and clear boundaries were also observed on both types of images. An arthroscopic examination revealed numerous white synovial, chondromate‐like tissues under the articular capsule, and D‐TGCT was diagnosed based on the histopathological results. One year after the surgery, the patient had good functional recovery and no tumor recurrence.

## 1. Introduction

A diffuse‐type tenosynovial giant cell tumor (D‐TGCT) is a rare, locally invasive synovial tumor that involves the synovial joints and tendon sheath [1, 2]. The 2020 World Health Organization (WHO) classification of soft tissue and bone tumors defines TGCT as a locally aggressive tumor [3, 4]. Most cases occur in female patients between the ages of 20 and 50 years [5]; reports of D‐TGCT in adolescents are limited. Of the reported adolescent cases, many involve the knee joint; rarely, the hip joint is affected [6]. Although most D‐TGCT is benign with a low mortality risk, joint destruction and repeated surgical intervention can severely affect the patient′s daily activities and quality of life [6–8]. Here, we report a case of unilateral hip joint D‐TGCT in a 14‐year‐old girl.

## 2. Case Report

A 14‐year‐old otherwise healthy girl complained of pain in the right hip with limited mobility for 2 years without a history of trauma. A local examination revealed mild tenderness of the femoral tuberosity of the right hip, and hip impingement and Faber test results were positive. Laboratory tests showed considerably elevated erythrocyte sedimentation rate and C‐reactive protein levels, but the levels of rheumatoid factor, antinuclear antibody, and other immune indicators were within the normal range. The total cholesterol and lipoprotein levels were slightly elevated, but the prealbumin level was low; other biochemical indicators were within the normal limits. Whole exome sequencing was performed at another hospital, which identified pathogenic mutations in the frizzled class receptor 4 (NM_012193.3:c.379C>T(p.Arg127Cys)) and apolipoprotein B (NM_000384.2:c.10579C>T(p.Arg3527Trp)) genes.

**Figure 1:**
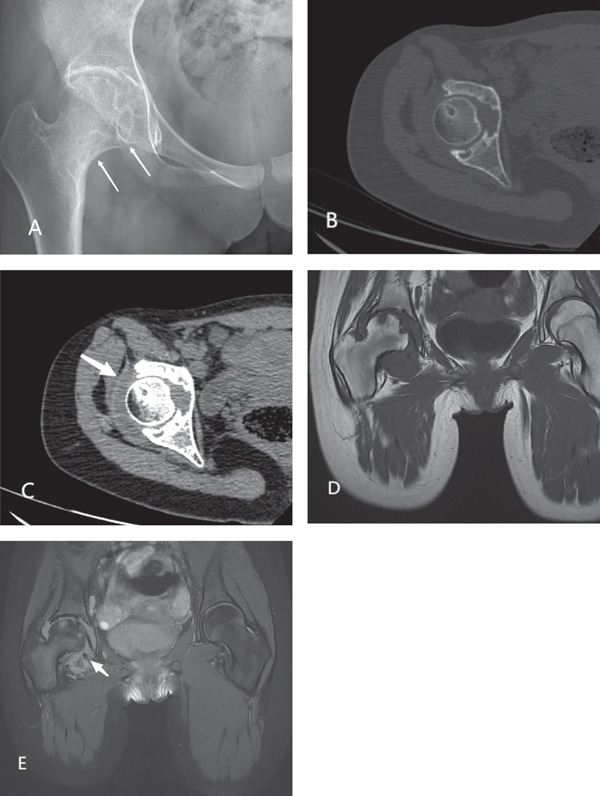
Pelvic imaging of the right hip joint of a 14‐year‐old girl with right hip pain and limited mobility. (A) X‐ray imaging reveals a bone defect in the right femoral head, femoral neck, and right hip joint, with a thin sclerotic edge at the boundary with normal bone. (B) Computed tomography imaging shows bone destruction at the joint edge, with a visible sclerotic zone at the junction with normal bone. (C) The synovial membrane around the right hip joint is unevenly thickened, presenting as a low‐density circular shadow surrounding the joint. (D) Magnetic resonance imaging shows thickening of the synovial membrane of the right hip joint with irregular edges, as well as low signal intensity on T1‐weighted images and high signal intensity on PDWI+FS images. (E) Small nodules with low signal intensity and clear boundaries are visible on T1‐weighted and T2 fat‐saturated images.

Pelvic radiography (Figure 1A) showed multiple circular bone defects under the right femoral head, femoral neck, and right hip joint surface with clear boundaries. Hardened edges were observed near the bone junction without obvious periosteal reactions or significant narrowing of the joint space. Computed tomography (CT) of the right hip joint (Figure 1B) showed irregular bone destruction at the joint edge, irregular sclerotic edges in the adjacent bone junction area, and uneven thickening of the synovial membrane around the right hip joint, which presented as low‐density circular shadows surrounding the right hip joint without signs of calcification or ossification. Magnetic resonance imaging (MRI) confirmed synovial membrane thickening of the right hip joint (Figure 1C–E), which manifested as a low signal on T1‐weighted images (WI) and a high signal with uneven edges on PDWI+FS images. Several small, nodular, low‐signal shadows with clear boundaries surrounding the thickened synovial membrane were also visible on T1WI and T2FS images.

**Figure 2:**
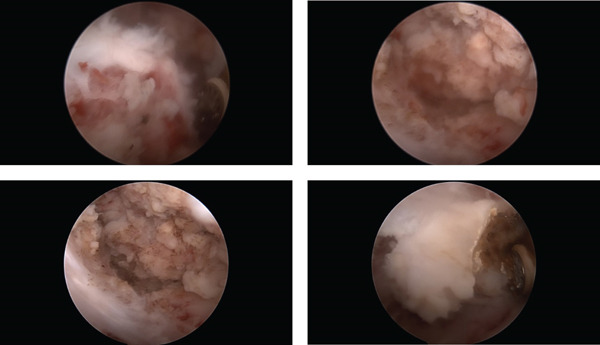
Arthroscopic images show a large amount of white synovial cartilage and tumor‐like tissue under the joint capsule that densely fills the joint capsule.

Based on the imaging data and test results, we suspected D‐TGCT of the hip joint that was invading the bone and suggested a pathological biopsy for confirmation. The patient and their family consented to the biopsy to clarify the pathological nature and surgical treatment to relieve the hip joint discomfort. MRI was used to assess the location and extent of tumor involvement, plan appropriate surgical procedures, and perform hip arthroscopies. We found that the articular cartilage was partially eroded with degeneration. We also found a large amount of white synovial cartilage tumor‐like tissue under the joint capsule that densely filled the joint capsule (Figure 2). Furthermore, part of the bone of the femoral head and neck was dented, and the tumor tissue adhered closely to the joint capsule and femoral neck, which was difficult to separate. Arthroscopically, we attempted to remove the tumor‐like tissue from the field of view and then completely stopped the bleeding.

**Figure 3:**
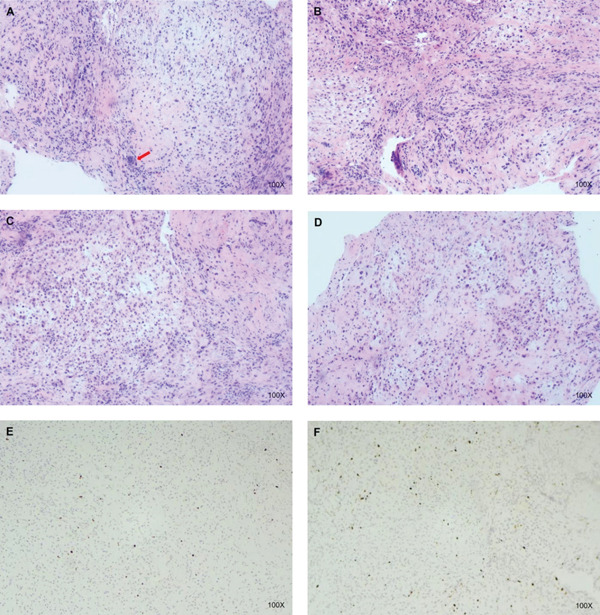
Examination of the right hip joint reveals hyperplasia of the synovial tissue, characterized by an abundance of synovial cells and the formation of fissure‐like structures. (A) One multinucleated giant cell was identified in our pathological sections. (B–D) Synovial monocyte proliferation was seen in sheets, with interstitial collagen fiber proliferation and inflammatory cell infiltration. (E–F) The immunohistochemical results were Ki‐67 (<1%). (A–F) Pictures were taken under an HE100 microscope.

**Figure 4:**
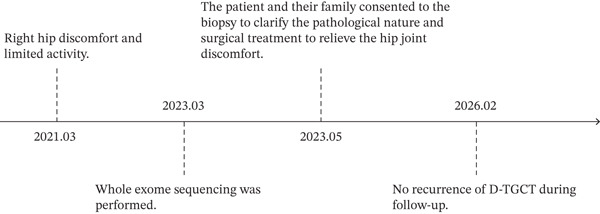
Timeline of the patient′s diagnosis with the relevant data about the treatment and follow‐up.

A postoperative pathological examination (Figure 3) revealed synovial tissue hyperplasia, interstitial collagen fiber tissue, small blood vessel hyperplasia, local congestion, scattered cells with nuclear deviation, lymphoid plasma cells, and neutrophil infiltration. The immunohistochemical results were: CK (–), EMA (–), CD56 (scattered+), CD68 (+), S‐100 (–), P63 (–), SATB2 (–), Ki‐67 (<1%+), and HMB45 (–). Pathological consultations from multiple hospitals revealed synovial tissue hyperplasia of the right hip joint, characterized by an abundance of synovial cells and the formation of fissure‐like structures. Additionally, interstitial sclerosis is present, accompanied by hyaline degeneration. Second‐generation sequencing conducted by the original unit did not identify any gene abnormalities associated with juvenile idiopathic arthritis. The observed lesions in the submitted tissue may be indicative of tenosynovial giant cell tumors (TGCT). The pathological diagnosis was D‐TGCT, which was confirmed by pathologists from Ruijin Hospital Affiliated with Shanghai Jiao Tong University, Shanghai Xinhua Hospital, and Taizhou Hospital. Figure 4 presents the timeline of the patient′s diagnosis, treatment, and follow‐up.

## 3. Discussion

D‐TGCT typically appears in major joints, such as the knee, ankle, hip, elbow, and shoulder [6, 9], with occasional cases in the spine [10]. The onset of D‐TGCT is typically slow, characterized by symptoms such as hip joint swelling, discomfort, and limited mobility. Furthermore, it is prone to recurrent attacks and does not worsen at night. A small number of patients with a history of trauma have lesions that can rapidly increase in the short term [11].

Radiography and CT manifestations of D‐TGCT of the hip joint are mostly diffuse and show uneven thickening of the synovial membrane around the hip joint, with the most visible bone destruction at the edge of the joint and visible sclerotic edges. In this case, the edge of the destroyed area was clear, and the bone destruction progressed slowly, without obvious periosteal reactions. Additionally, there were no obvious signs of calcification in the adjacent soft tissues, and the joint space was generally not considerably narrowed. Some researchers have suggested that the hip joint capsule is tightly attached and difficult to expand; thus, when disease occurs, the pressure inside the joint considerably increases, leading to bone destruction [3, 6, 11].

In the hip joint, D‐TGCT manifests on plain MRI as a low signal on T1WI and an uneven low signal on T2WI. In some cases, lesions appear as cystic areas with low T1WI and high T2WI signals, exhibiting moderate to significant enhancement. However, the low‐signal areas of some lesions on T2WI are not clearly displayed on a T2FS sequence. Notably, a few lesions have mild or inconspicuous bone destruction on radiography and CT, and the T2WI MRI images have low signal intensity, which can easily lead to a misdiagnosis.

The MRI manifestations of D‐TGCT are classified into three types based on a cystic change in the lesion and its proportion. Type I is more common; solid components are the primary manifestation, with minimal or no fluid accumulation or cystic change. Type II is characterized by the coexistence of solid and cystic components of the lesion, with little difference in the proportion between the two. Type III is relatively rare, with cystic components as the main lesion, and diffuse mild thickening or local nodular thickening of the synovium [11]. The primary manifestation in this case was diffuse thickening of the synovial membrane with locally visible nodular abnormal signals. In cases of D‐TGCT affecting the hip joint, bone erosion and degeneration of subchondral bone cysts are frequently observed. Consequently, we classified the D‐TGCT in this case as Type III. Unfortunately, enhanced magnetic resonance examination was not performed. The MRI manifestations of Types I and II D‐TGCT have high specificity and are relatively easy to diagnose, whereas Type III D‐TGCT is prone to misdiagnosis. Therefore, understanding the imaging characteristics of various types of D‐TGCT is crucial for comprehensively understanding this disease and reducing misdiagnoses and missed diagnoses.

D‐TGCT of the hip joint can be clearly diagnosed by X‐ray and CT in cases with obvious bone destruction and synovial thickening. In cases of mild bone destruction that are not sensitive to X‐rays, CT is advantageous for displaying fine structures. Therefore, in cases where plain radiographs suggest bone destruction, CT can be performed for further clarification. MRI is uniquely advantageous for detecting specific ferroflavin signals and determining the extent of lesions [6, 12]. PDWI+FS is often used in joint imaging: It displays articular cartilage, ligaments, and other structures more clearly, and can better observe the shape, thickness, and presence of damage of the cartilage. Due to its high signal‐to‐noise ratio and delicate image, it can detect some tiny lesions. Display has certain advantages. T2WI has a better effect on displaying the areas where fluid accumulates in the body, is more sensitive to displaying edema, inflammation, cysts, and other lesions, and can more clearly display the scope and degree of lesions. D‐TGCT is prone to postoperative recurrence, so accurate and complete preoperative visualization of the lesion is essential for selecting the appropriate surgical method and determining the surgical scope. If no obvious bone destruction is observed on plain radiographs or CT scans, a walking MRI examination can be performed to assist in the diagnosis and understand the extent of the lesion.

The patient in this case was diagnosed with juvenile idiopathic arthritis, and unexpectedly, *FZD4* and *APOB* mutations were identified using whole exome sequencing. The patient reported having a retinal hole and was treated accordingly, consistent with the detected *FZD4* variant [13]. Additionally, we unexpectedly identified a variant of APOB associated with type B hypercholesterolemia [14, 15]. The gene responsible for autosomal dominant hypercholesterolemia type B (OMIM:144010) is APOB, situated at Chromosomal Location 2:21229161. This condition follows an autosomal dominant inheritance pattern, wherein heterozygous pathogenic variants in the APOB gene can lead to the manifestation of the disease. Studies have shown that inherited hypercholesterolemia leads to the excessive accumulation of cholesterol in tissues other than the liver. TGCT cell populations are diverse and include round stromal cells, multinucleated giant cells, and hemosiderin‐rich lipid foam cells. Therefore, lipid foam cells are proposed to be the main pathological entity of TGCT, and lipid metabolism disorders are considered the leading causes of the disease [16]. TGCT is postulated to be a secondary inflammatory reaction resulting from a local lipid metabolism disorder, which is caused by trauma from a cholesterol metabolism disorder; however, there is often no conclusive evidence. In this adolescent patient, the total cholesterol and lipoprotein levels were elevated, despite a body mass index of 16.2 and a prealbumin concentration of 14.4 mg/dL, which was likely influenced by the *APOB* variant. This is the first study to demonstrate a genetic link between a cholesterol metabolism disorder caused by an *APOB* variant and the occurrence of TGCT.

The patient in this case was 14 years old. Therefore, synovial hyperplasia and erosion of the bony tissue in the affected hip impaired limb development to a certain extent. Synovial erosion was considered; however, the pathological characteristics were unknown. There are clear indications for surgical clearance and pathological examinations. If open surgery is performed, the focal tissue can be thoroughly cleaned [6]. However, the surgical trauma is severe, and round ligament injury may cause early necrosis of the femoral head. The greatest advantage of arthroscopic surgery is that it is minimally invasive with less trauma and faster recovery [17]. However, there is a possibility of early recurrence owing to incomplete cleaning. Based on the preoperative imaging and various other test results, our patient was diagnosed with D‐TGCT, increasing the possibility of a benign tumor. Since D‐TGCT is a locally invasive, nonmetastatic tumor, the main risks of surgery are recurrence and joint injury. The treatment principle is to remove the tumor as thoroughly as possible and preserve limb function to the maximum extent. Radical surgery combined with radiotherapy can be used in cases of multiple recurrences. Abnormalities in the CSF1 gene are frequently observed in TGCT. The chromosomal translocation t(1;2)(p13;q37) results in the CSF1:COL6A3 fusion, which represents the most prevalent fusion variant. Some patients do not exhibit CSF1 gene fusions or present with genetic abnormalities unrelated to the CSF1 gene [18]. We also suggested RNA next‐generation sequencing for colony‐stimulating factor 1 (i.e., CSF1) rearrangement after surgery; For unresectable TGCT, treatment with inhibitors of the CSF1/CSF1R pathway is recommended. However, the patient refused. After 6 months, the patient′s range of motion in the right hip improved significantly, and the pain was relieved; importantly, D‐TGCT did not recur.

To our knowledge, this is the first documented instance of a pathogenic variant in the *APOB* gene connected to D‐TGCT. Additionally, this is one of the few reported cases of D‐TGCT in adolescent hips. Our patient′s specific imaging manifestations could have easily led to a misdiagnosis. Therefore, understanding the imaging characteristics of various types of D‐TGCT is crucial for comprehensively understanding this disease and, consequently, reducing misdiagnoses and missed diagnoses. We will continue to follow‐up with this patient in the future.

## Author Contributions

Yujia Wang and Shaohua Zhang contributed equally to this work.

## Funding

This work was supported by the Social Development Science and Technology Plan Project of Taizhou City (25ywb17).

## Ethics Statement

Written informed consent was obtained from the individuals for the publication of any potentially identifiable images or data included in this article.

## Conflicts of Interest

The authors declare no conflicts of interest.

## Data Availability

The data that support the findings of this study are available from the corresponding authors upon reasonable request.
